# Extracellular nucleotides inhibit growth of human oesophageal cancer cells via P2Y_2_-receptors

**DOI:** 10.1038/sj.bjc.6600100

**Published:** 2002-02-12

**Authors:** K Maaser, M Höpfner, H Kap, A P Sutter, B Barthel, B von Lampe, M Zeitz, H Scherübl

**Affiliations:** Medical Clinic I, Gastroenterology/Infectious Diseases/Rheumatology, Benjamin Franklin Clinics, Free University of Berlin, Hindenburgdamm 30, 12200 Berlin, Germany

**Keywords:** cancer, proliferation, apoptosis, purinergic receptor, calcium signalling

## Abstract

Extracellular ATP is known to inhibit growth of various tumours by activating specific purinergic receptors (P2-receptors). Since the therapy of advanced oesophageal cancer is unsatisfying, new therapeutic approaches are mandatory. Here, we investigated the functional expression and potential antiproliferative effects of P2-purinergic receptors in human oesophageal cancer cells. Prolonged incubation of primary cell cultures of human oesophageal cancers as well as of the squamous oesophageal cancer cell line Kyse-140 with ATP or its stable analogue ATPγS dose-dependently inhibited cell proliferation. This was due to both an induction of apoptosis and cell cycle arrest. The expression of P2-receptors was examined by RT-PCR, immunocytochemistry, and [Ca^2+^]_i_-imaging. Application of various extracellular nucleotides dose-dependently increased [Ca^2+^]_i_. The rank order of potency was ATP=UTP>ATPγS>ADP=UDP. 2-methylthio-ATP and α,β-methylene-ATP had no effects on [Ca^2+^]_i_. Complete cross-desensitization between ATP and UTP was observed. Moreover, the phospholipase C inhibitor U73122 dose-dependently reduced the ATP triggered [Ca^2+^]_i_ signal. The pharmacological features strongly suggest the functional expression of G-protein coupled P2Y_2_-receptors in oesophageal squamous cancer cells. P2Y_2_-receptors are involved in the antiproliferative actions of extracellular nucleotides. Thus, P2Y_2_-receptors are promising target proteins for innovative approaches in oesophageal cancer therapy.

*British Journal of Cancer* (2002) **86**, 636–644. DOI: 10.1038/sj/bjc/6600100 www.bjcancer.com

© 2002 Cancer Research UK

## 

ATP and related compounds are widespread transmitters for extracellular communication in many cell types. By coupling to specific purinergic receptors ATP is involved in a large variety of cellular functions (Ralevic and Burnstock, 1998). The classification of P2 purinergic receptors, first suggested by Burnstock in the late seventies (Burnstock, 1978) distinguished between two major classes of P2-purinoceptors, first ionotropic P2-receptors (P2X), which are ligand-gated receptors containing an intrinsic ion channel, and second metabotropic P2-receptors (P2Y), which belong to the superfamily of G-protein coupled receptors. This classification has recently been expanded (Barnard *et al*, 1994; Fredholm *et al*, 1997) to accommodate the results of cloning studies which revealed the existence of multiple subclasses of P2Y purinoceptors. While ionotropic P2X-receptors are mainly expressed in the nervous system, platelets and in smooth muscle cells, metabotropic P2Y-receptors are distributed in a large variety of tissues, including epithelial tissues of the gastrointestinal system (Dubyak and el-Moatassim, 1993; Roman and Fitz, 1999). P2Y-receptors mainly couple to G_q/11_-proteins to activate phospholipase C. Upon activation phosphatidylinositolbisphosphate (PIP_2_) is hydrolyzed leading to the generation of IP_3_ (inositoltrisphosphate), which triggers calcium release from intracellular stores (SR/ER) (Lytton *et al*, 1991). Depletion of intracellular calcium stores in turn induces calcium influx across the plasma membrane (Lytton *et al*, 1991). This Ca^2+^-influx mechanism, which depends on an initial elevation of [Ca^2+^]_i_, has been referred to as ‘calcium release’ activated calcium influx’ or ‘capacitative calcium entry’ (Berridge, 1995).

ATP is known to inhibit cancer growth in various tumour models (Rapaport, 1990; Haskell *et al*, 1996; Correale *et al*, 1997; Ishikawa *et al*, 1997). ATP-induced apoptosis was in the past considered to be mediated by ionotropic P2-receptors only (Fredholm *et al*, 1994; Bronte *et al*, 1996; Correale *et al*, 1997). However, there is growing evidence that by altering intracellular calcium concentrations metabotropic P2-receptors may be involved in both growth inhibition and programmed cell death (Fang *et al*, 1992; Duncan *et al*, 1996; McConkey and Orrenius, 1997; Höpfner *et al*, 2001).

Oesophageal cancers have not been studied in this respect so far. Therefore we investigated the P2-receptor-mediated, cell cycle arresting and apoptosis inducing effects of extracellular nucleotides in oesophageal cancer cells.

## MATERIALS AND METHODS

### Materials

Fura-2/AM, gramicidin, ATP, ATPγS, UTP, ADP, UDP, 2-methylthio-ATP (2MeSATP), α,β-methylene-ATP (α,β-meATP) and adenosine were purchased from Sigma Chemicals Co. (München, Germany). SBFI/AM and pluronic-F127 were purchased from Molecular Probes (Eugene OR, USA). RPMI 1640 was obtained from Biochrom-Seromed KG (Berlin, Germany). All stock solutions except from fura-2/AM, SBFI/AM and adenosine (solubilized in DMSO) were prepared in water and stored in aliquots of appropriate size at −20°C until use.

### Cell culture

The human oesophageal squamous carcinoma cell line Kyse-140 (Shimada *et al*, 1992) was maintained in standard RPMI 1640 medium, complemented with 10% foetal calf serum and was kept in an incubator (37°C, 5% CO_2_ humidified atmosphere).

Primary cell cultures of histologically verified oesophageal squamous cell carcinomas were established from endoscopic biopsies of seven oesophageal cancer patients (three female, four male patients, age: 52–69 years). The human tumour material was used according to the standards set by the Ethical Committee of the Benjamin Franklin University Hospital, Free University of Berlin, Germany. Preparation was performed by mechanical dissection using an automated disaggregation system (Medimachine; Becton Dickinson, Heidelberg, Germany) as described elsewhere in detail (Maaser *et al*, 2001). The isolated human oesophageal carcinoma cells were maintained in nutrient medium based on Earle's 199-medium (Biochrom, Berlin, Germany) complemented with 20% foetal calf serum, 2 mM L-glutamine, 2% (v/v) Biotect protective-medium (Biochrom, Berlin, Germany), penicillin (100 U ml^−1^), streptomycin (100 μg ml^−1^), amphotericin B (1% v/v) and were kept in an incubator (37°C, 5% CO_2_ humidified atmosphere). Half of the medium was changed every second day. Cells remained in culture for at least 2 days before the experiments were carried out. Squamous epithelial origin of the isolated primary culture cells was confirmed by immunostaining with a FITC-labelled cytokeratin antibody (cytokeratin/FITC clone MNF 116, DAKO, Hamburg, Germany).

### Cell proliferation assay

Antiproliferative effects of a sustained application of P2-receptor agonists on human oesophageal carcinoma cells were studied by performing proliferation assays according to the crystal violet method (Gillies *et al*, 1986). In brief, Kyse-140 cells were seeded on 96-well plates at a density of 5000 cells per well. ATP, ATPγS, 2MeSATP or adenosine were added in concentrations from 100 to 500 μM. If stated, cells were additionally treated with 10 or 100 μM of the ecto-ATPase inhibitor ARL 67156 (6-N,N- diethyl-β-γ-dibromomethylene-D-adenosine-5′-triphosphate trisodium; RBI, Natick, MA, USA) which was given 1 h before as well as during nucleotide incubation. Each concentration group consisted of 10 wells. The incubation medium was changed daily. Cells of each well were washed with phosphate-buffered saline (PBS) and fixed with 100 μl glutaraldehyde (1% v/v) in PBS for 15 min at room temperature. After another washing step cells were stained with 0.1% crystal violet in PBS for 30 min at room temperature. The unbound dye was removed by washing with H_2_O for 30 min. Crystal violet which had absorbed onto the cells was solubilized with 100 μl Triton X-100 (0.2%) in PBS for at least 24 h at 37°C. The crystal violet coloured solution was spectrophotometrically measured at 570 nm using an ELISA-Reader. In the range between 500 to 100 000 cells per well the measured extinction was linear to the number of cells.

### Cell cycle analysis

Cell cycle analysis was performed by using the method of Vindelov and Christensen (1990). 5×10^5^ cells per well were cultured for 24 h and then exposed to the respective nucleotide for another 24 h. Cells were trypsinized, washed, and the nuclei were isolated using CycleTest PLUS DNA Reagent Kit (Becton Dickinson, Heidelberg, Germany). DNA was stained with propidium iodide according to the manufacturers' instructions. The DNA content of the nuclei was detected by flow cytometry and analyzed using CellFit software (Becton Dickinson, Heidelberg, Germany).

### Caspase-3 activity assay

Cells were incubated with medium containing ATP or ATPγS at concentrations from 50 to 500 μM for 48 h, washed twice with PBS, and stored at −80°C until use. Approximately 10^6^ cells were lysed with 500 μl lysis buffer (10 mM Tris-HCl, 10 mM NaH_2_PO_4_/Na_2_HPO_4_, 130 mM NaCl, 1% Triton X-100, 10 mM NaPP_i_, pH 7.5) and total protein content was quantified using the BCA protein assay kit (Pierce, Rockford, IL, USA). Caspase-3 activity was calculated from the cleavage of the fluorogenic substrate DEVD-AMC (Nicholson *et al*, 1995). In brief, 100 μl cell lysate containing 500 μg ml^−1^ protein was incubated with 100 μl substrate solution (2 μg caspase-3 substrate AC-DEVD-AMC, 20 mM HEPES, 10% glycerol, 2 mM DTT, pH 7.5) for 1 h at 37°C. Cleavage of DEVD-AMC was measured with a VersaFluor fluorometer (Biorad, Munich, Germany) using a 360 nm excitation and a 460 nm emission wavelength.

### TUNEL assay

Determination of apoptosis specific DNA strand breaks was performed by terminal deoxynucleotidyl transferase-mediated dUTP nick-end labelling (TUNEL) assay (Li *et al*, 1996) according to the manufacturers' instructions (Roche, Mannheim, Germany). Cells were fixed with 4% paraformaldehyde for 1 h at room temperature, washed once with 200 μl 1% BSA in PBS, followed by permeabilization with 0.1% Triton X-100 in 0.1% sodium citrate solution for 2 min on ice. After washing with 200 μl 1% BSA in PBS, cells were stained with 50 μl TUNEL reaction mixture (Roche, Mannheim, Germany) for 1 h at 37°C in a humidified atmosphere in the dark. After two additional washing steps, nuclear fluorescence was analyzed using a Zeiss Axioskop-2 microscope.

### Reverse transcriptase chain reaction (RT–PCR)

RNA isolation, reverse transcription and PCR reactions were carried out as described elsewhere in detail (Glassmeier *et al*, 1998). Total RNA was isolated from cells using the RNeasy Kit (Qiagen, Valencia, CA, USA) according to the manufacturers' instructions. To eliminate any possible contamination with genomic DNA, RNAs were treated with 1 U DNAse I (Gibco, Karlsruhe, Germany) per μg RNA for 15 min at room temperature. Furthermore, possible contamination with genomic DNA was excluded by control experiments omitting the reverse transcriptase. Purified RNA was reverse transcribed into cDNA using the oligo-dT-primers and the SuperScript Preamplification-Kit (Gibco, Karlsruhe, Germany). PCR reactions were carried out in a total volume of 50 μl containing 400 nM of each primer, 200 μM of each dNTP (Pharmacia, Uppsala, Sweden), 50 mM KCl, 1.5 mM MgCl_2_, 10 mM Tris, and 1 Unit Taq-polymerase (Pharmacia, Uppsala, Sweden). PCR was performed in a Peltier-thermocycler (PTC-200, MJ-Research, USA) at the following conditions: Initial heating to 95°C for 5 min, then 30 cycles at 95°C for 30 s, 60°C for 30 s for P2X- and P2Y_6_-primers, or 70°C for 30 s for P2Y_1,2,4_-primers, or 63°C for 1 min for β-actin primers, respectively, and 72°C for 1 min, followed by a final elongation step of 7 min at 72°C. Amplification of the cDNA sequences encoding for the tested human P2-receptor subtypes was performed using specific and established primer sequences (Schöfl *et al*, 1999; Yamamoto *et al*, 2000; Höpfner *et al*, 2001) as shown in Table 1

**Table 1:**
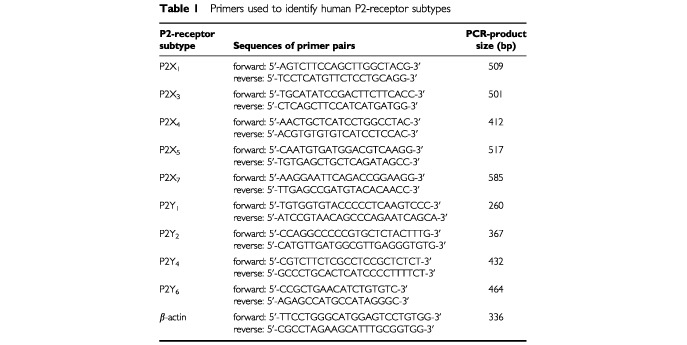
Primers used to identify human P2-receptor subtypes

. The specificity of the cDNA products was additionally controlled by direct sequencing which was performed by Invitek Sequencing Service GmbH, Berlin-Buch, Germany. As a control for the amount of cDNA, RT–PCR was performed using β-actin primers (Table 1).

### Immunofluorescence labelling and fluorescence microscopy

Cells were washed with PBS, fixed by incubation with 4% paraformaldehyde in PBS for 30 min at room temperature, and washed again with PBS. Cells were then incubated for 3 h at room temperature with 5 μg ml^−1^ polyclonal rabbit-anti-human P2Y_2_ antibody, which was kindly provided by Dr BK Kishore (Kishore *et al*, 2000), or with 5 μg ml^−1^ polyclonal rabbit-anti-human P2Y_4_ antibody Ab-1 (Oncogene, Cambridge, CA, USA). Thereafter cells were washed twice with PBS and incubated with 4 μg ml^−1^ secondary Alexa™ 488-labelled goat-anti-rabbit IgG antibody (Molecular Probes, Eugene, OR, USA) for 1 h at room temperature. Fluorescence and transmission images were obtained using a Zeiss Axioskop-2 microscope.

### Measurement of intracellular calcium and sodium

Kyse-140 cells were grown on 25 mm glass coverslips coated with poly-*L*-lysine for improved attachment. For measurement of intracellular calcium cells were loaded with the fluorescent dye fura-2/AM (5 μM) for 45 min at room temperature in a bath solution containing: 130 mM NaCl, 5.4 mM KCl, 1 mM CaCl_2_, 1 mM MgCl_2_, 10 mM glucose and 10 mM HEPES, adjusted to pH 7.3 with NaOH. The constituents of Ca^2+^-free solutions were the same as the bathing solution described above except that CaCl_2_ was omitted and 0.1 mM EGTA was added to eliminate possible Ca^2+^-contaminations of the other constituents. Fluorescence measurements were performed at room temperature with a digital imaging system of T.I.L.L. Photonics (München, Germany) (Glassmeier *et al*, 1998; Höpfner *et al*, 1998). Fura-2 fluorescence was excited alternatively at 340 and 380 nm with a monochromator provided by a 75 W xenon lamp. Cellular fluorescence was filtered through a 510 nm band pass filter. Images were digitalized and analyzed by FUCAL 5.12c software of T.I.L.L. Photonics. Ratio images were generated either at 1.5 or 2 s intervals. To compensate for background noise illumination of a cell-free area was subtracted. The calculation of the fura-2 signal was done as described in detail elsewhere (Morgan and Thomas, 1999). To calculate [Ca^2+^]_i_ the equation of Grynkiewicz *et al* (1985) was used. The data are mainly represented as changes in 340/380 nm (ΔF340/F380 nm) fluorescence ratio, which is proportional to [Ca^2+^]_i_. Unless representative single tracings are shown, the results are given as means±s.e.m.

Changes in intracellular sodium concentration were monitored by loading the cells with the fluorescent dye SBFI/AM (10 μM) for 2 h at 37°C, 5% CO_2_ humidified atmosphere in the same bath solution as described above. For better dispersion of the dye in the loading medium the nonionic and nondenaturing detergent pluronic F-127 (0.04% (w/v)) was added. To remove extracellular remainders of SBFI/AM, the cells were washed three times in bath solution. Then the samples were kept in bath solution for at least 20 min to ensure complete intracellular de-esterafication of the dye. Fluorescence recordings with SBFI-loaded cells were performed under the same conditions as described above for fura-2, except from the excitation wavelength of the Na^+^-bound dye which was 344 nm. The data are represented as changes in 344/380 nm (ΔF344/F380 nm) fluorescence ratio, which is proportional to [Na^+^]_i_.

### Statistical analysis

Comparison of multiple means was performed with nonparametric ANOVA. Comparison of individual drug treatments to control treatments was performed using an unpaired, two-tailed Mann–Whitney *U*-test for proliferation and caspase-3 activity experiments. Data are expressed as mean percentage of control±s.e.m. For cell cycle analysis the unpaired student *t*-test was used. *P* values were considered to be significant at <0.05.

## RESULTS

### Growth inhibitory effects of extracellular nucleotides on oesophageal cancer cells

**Figure 1:**
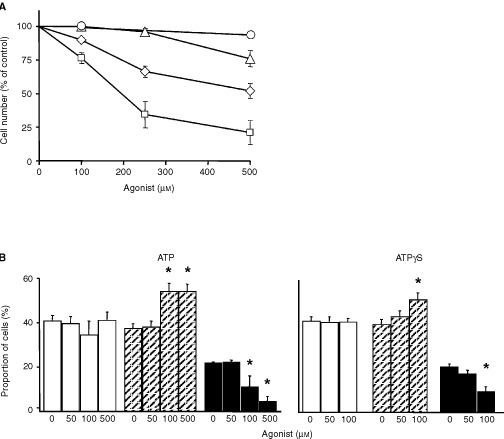
Antiproliferative and cell cycle effects of nucleotides. (**A**) Extracellular ATP (⋄) and ATPγS (□) significantly decreased the cell number of Kyse-140 cells after 3 days of incubation in a dose-dependent manner (*P*<0.05 for 100–500 μM). In contrast, 2MeSATP (○) or adenosine (Δ) showed no or minor effects. The respective antiproliferative effects are given as the percentage of cells of the untreated controls (=100%). Means±s.e.m. of four independent experiments are shown. (**B**) After 24 h of incubation both ATP and ATPγS altered the proportion of cells in the G1/G0-phase (white columns), S-phase (hatched columns), and G2/M-phase (black columns) of the cell cycle. Means±s.e.m. of six independent experiments are shown.

ATP is known to inhibit cancer growth in various tumour models (Agteresch *et al*, 1999). However, its role in growth control of human oesophageal cancer remains elusive. To investigate putative growth modulating effects of extracellular nucleotides in oesophageal carcinoma cells, cell proliferation assays were performed with Kyse-140 cells. For these experiments ATP, the hydrolysis resistant ATP derivative ATPγS, adenosine, and 2MeSATP were used ( Figure 1A

). Both ATP and ATPγS dose-dependently decreased cellular proliferation. The poorly hydrolysable ATPγS was even more effective (IC_50_=234±51 μM after 72 h of incubation) than ATP (IC_50_=450±31 μM). In contrast, 2MeSATP failed to inhibit the growth of Kyse-140 cells even at a concentration of 500 μM. Adenosine, the major breakdown product of ATP, showed no effects on cellular proliferation up to 250 μM. Only at the high adenosine concentration of 500 μM, mimicking the unlikely complete degradation of the maximal ATP-dose of 500 μM to adenosine, a growth inhibitory effect by adenosine of 24±6% was observed, presumably mediated by an activation of adenosine specific P1-receptors, which are ubiquitously expressed (Fang *et al*, 1992; Vandewalle *et al*, 1994). However, the adenosine-evoked effect is about 50% less than those of ATP at the same concentration of ATP (52±6%). This means that even in case of a complete hydrolysis of ATP to adenosine, the antiproliferative effects of an ATP treatment can only be partially explained by an involvement of adenosine-mediated effects.

Ecto-ATPases are membrane bound enzymes which dephosphorylate ATP, thereby terminating the stimulatory effects of ATP on P2-receptors. It has been shown by others that in several tissues the activation of ecto-ATPases themself is capable of altering intracellular second-messengers (e.g. NO) thereby also affecting cell growth (Sneddon *et al*, 2000). To exclude a possible contribution of ecto-ATPases in the regulation of cell growth, the effects of the ecto-ATPase inhibitor ARL 67156 (10–100 μM) were investigated. However, even 100 μM of the ecto-ATPase inhibitor ARL 67156 showed no significant influence on proliferation of Kyse-140 cells, thus excluding a contribution of ecto-ATPase signalling to the observed growth inhibitory effects (data not shown).

### Effects of extracellular nucleotides on the cell cycle

To investigate if the observed growth inhibiting effects of extracellular nucleotides on oesophageal cancer cells were caused by cell cycle arrest, cell cycle analysis was performed. After sustained incubation of Kyse-140 cells with either ATP or ATPγS for 24 h a dose-dependent increase of the proportion of cells in the S-phase of the cell cycle was observed, indicating S-phase delay of the cells (Figure 1B). To exclude a possible contribution of breakdown products of ATP, in particular of adenosine, cell cycle analysis was performed with adenosine. In accordance with the proliferation data, adenosine (50–500 μM) did not alter the regulation of the cell cycle (data not shown).

### Apoptotic effects of extracellular nucleotides

**Figure 2:**
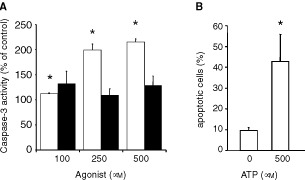
Apoptotic effects of extracellular nucleotides. (**A**) ATP (white columns) but not ATPγS (black columns) dose-dependently induced caspase-3 activity in Kyse-140 cells after 48 h of incubation. Caspase-3 activity was measured as the cleavage of the fluorogenic substrate DEVD-AM. Means±s.e.m. of four independent experiments are given as the percentage of fluorescence compared to untreated control. (**B**) Nucleic DNA fragmentation of human oesophageal primary culture cells was determined using TUNEL assay. Incubation with 500 μM ATP for 48 h induced a significant increase of the proportion of nucleic fluorescence staining. The proportions of positively stained cells are shown as means±s.e.m. of three independent preparations.

To check whether the antiproliferative effects of ATP and ATPγS, respectively, were caused not only by cell cycle arrest but also by an induction of programmed cell death, the activation of caspase-3, a key-enzyme in the process of apoptosis, was investigated in Kyse-140 cells. After 48 h of incubation ATP dose-dependently induced an increase of caspase-3 activity. With 500 μM ATP the caspase-3 activity was more than doubled compared to the untreated control ( Figure 2A

). ATP-induced caspase-3 activity was not due to a generation of adenosine, since adenosine (50–500 μM) failed to stimulate caspase-3 activity in Kyse-140 cells (data not shown). ATPγS did not affect caspase-3 activity (Figure 2A). Three to 72 h of incubation with rising concentrations of ATPγS (50–500 μM) did not evoke significant effects on caspase-3 activity. To investigate a possible contribution of ecto-ATPase-dependent generation of extracellular P_i_ by degradation of ATP, which has already been shown to be involved in ATP-induced apoptosis (Zoeteweij *et al*, 1993), we pre-incubated the cells with 100 μM of the ecto-ATPase-inhibitor ARL 67156. Under these conditions the ATP (500 μM for 48 h) evoked induction of caspase-3 activity decreased by 18±3.5% while ARL 67156 alone even increased caspase-3 activity by 28±16 %. The data suggest that ecto-ATPase dependent generation of extracellular P_i_ is involved in ATP-induced apoptosis of oesophageal cancer cells.

In primary oesophageal cancer cells induction of apoptosis by ATP was investigated by end-labelling of DNA with fluorescein-dUTP (TUNEL assay). Fluorescence microscopy of primary cell cultures of oesophageal cancers treated with 500 μM ATP for 48 h revealed an increase in apoptosis specific DNA strand breaks of 32.7±13% above the untreated control which showed an average proportion of apoptotic cells of 9.6±1.4%. (Figure 2B).

### Expression of P2-receptor mRNA and protein

**Figure 3:**
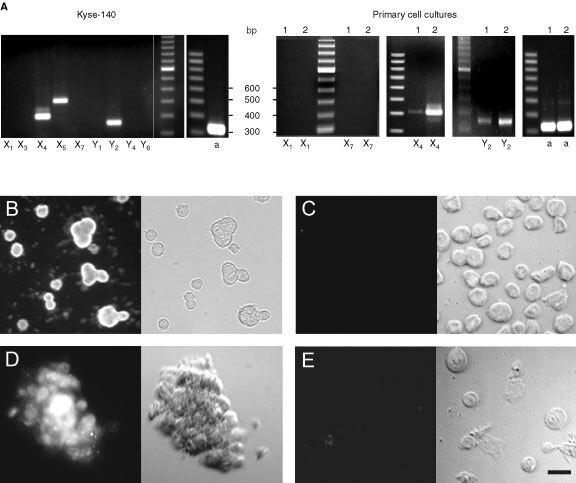
Expression of P2-receptor subtypes. (**A**) Detection of mRNAs of ionotropic and metabotropic P2-receptor subtypes and of β-actin (a) in Kyse-140 cells as well as in oesophageal primary cell cultures by reverse transcriptase polymerase chain reaction (RT–PCR). Transcripts of two representative primary cell cultures (1 and 2) out of four are shown. Amplified cDNA was visualised by gel electrophoresis on an ethidium-bromide-stained agarose gel. (**B**–**E**) Kyse-140 cells (**B**,**C**) or oesophageal primary culture cells (**D**,**E**) were incubated with either specific P2Y_2_ antibody (**B**,**D**) or P2X_4_ antibody (**C**,**E**) and subsequently with secondary fluorescence-labelled antibody. Left side: fluorescence images, right side: corresponding transmission light images. Bar=20 μM.

**Figure 4:**
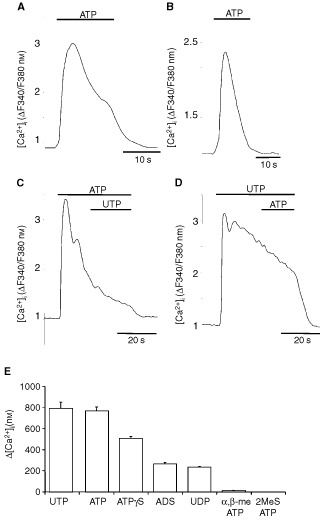
Functional expression of P2-receptors. (**A**) Biphasic increase of [Ca^2+^]_i_ after ATP application (100 μM) in Ca^2+^-containing bath solution. (**B**) Monophasic [Ca^2+^]_i_ rise after ATP application (100 μM) in Ca^2+^-free solution. (**C**) Application of UTP (100 μM) during prolonged ATP stimulation (100 μM) failed to elicit any further response. (**D**) Application of ATP (100 μM) during prolonged UTP stimulation (100 μM) did not cause any further rise of [Ca^2+^]_i_. The [Ca^2+^]_i_ is given as the fluorescence ratio (ΔF340/F380 nm) of the Ca^2+^-sensitive dye fura-2. Representative single-cell tracings out of three independent experiments with 20 or more cells per incubation are shown. (**E**) Different nucleotides in equimolar concentration (100 μM) induced distinct increases of [Ca^2+^]_i_ in KYSE-140 cells measured under [Ca^2+^]_e_-containing conditions. Values are given as the peak increase above basal [Ca^2+^]_i_ of resting cells. The basal level of resting cells amounted to 58±7 nM. Means±s.e.m. of 44 to 98 cells per nucleotide are shown.

To elucidate which P2-receptor subtype(s) may be responsible for the growth inhibiting and apoptotic effects of extracellular nucleotides, the mRNA expression of ionotropic P2-receptors (P2X_1_, P2X_3_, P2X_4_, P2X_5_, and P2X_7_) and metabotropic P2-receptors (P2Y_1_, P2Y_2_, P2Y_4_, and P2Y_6_) was investigated by RT–PCR. Kyse-140 cells expressed mRNAs of P2X_4_-, P2X_5_-, and P2Y_2_-receptors ( Figure 3A

). Interestingly, no mRNA-expression of P2X_1_- or P2X_7_-receptors, which are mainly implicated in the induction of apoptosis (Chow *et al*, 1997; Di Virgilio *et al*, 1998; Ferrari *et al*, 1999) was observed. The mRNA of P2Y_2_- and P2X_4_ receptors was also detected in biopsies of human squamous cancers of the oesophagus, while transcripts of P2X_1_ and P2X_7_ receptors were also missing in the mRNA of oesophageal cancer biopsies ( Figure 4A

). The specificity of PCR was confirmed by direct sequencing of transcripts (data not shown).

However, using immunocytochemistry only a P2Y_2_-receptor-specific fluorescence was detected in the membrane of either Kyse-140 and primary culture cells (Figure 3B,D), whereas neither Kyse-140 cells nor primary cell culture preparations showed any specific staining for P2X_4_ receptors (Figure 3C,E). Thus it is likely, that despite the mRNA expression of P2X_4_ receptors no translation into P2X_4_ protein occurs in the cells.

### Expression of functional P2-receptors

To evaluate which P2-receptor subtypes mediate apoptosis and cell cycle arrest in oesophageal cancer cells, subtype-specific expression of functional P2-receptors was investigated by measuring changes in [Ca^2+^]_i_. In Ca^2+^-containing bath solution, ATP (100 μM) induced a rapid biphasic increase of [Ca^2+^]_i_. An initial transient [Ca^2+^]_i_ peak was followed by a sustained plateau-phase (Figure 4A). Regardless of the omission of Ca^2+^ from the extracellular bath, ATP still induced a rapid but now only transient increase of [Ca^2+^]_i_ (Figure 4B). This observation suggests that the initial [Ca^2+^]_i_ peak was caused by the release of Ca^2+^ from intracellular stores, whereas the plateau-phase occurring under [Ca^2+^]_e_-containing conditions was due to transmembraneous Ca^2+^-influx. The biphasic [Ca^2+^]_i_ response to stimulation with ATP is a typical feature of metabotropic P2Y-receptors. Similar to ATP, UTP induced a comparable increase of [Ca^2+^]_i_ (Figure 4D), indicating the expression of purine and pyrimidine-sensitive receptors in oesophageal cancer cells. Pre-treatment of the cells with 100 μM of either ATP or UTP prevented a second rise in [Ca^2+^]_i_ in response to a consecutive stimulation with the respective other nucleotide (Figure 4C,D). This complete homologous cross-desensitization between ATP and UTP indicates that both nucleotides were competitively acting on a common binding site. Since only one metabotropic P2Y-receptor equipotently activated by either ATP or UTP, is known so far, these data strongly suggest the functional expression of P2Y_2_-receptors in Kyse-140 cells.

**Figure 5:**
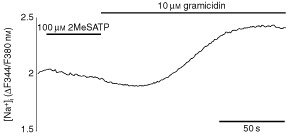
Effect of 2MeSATP on [Na^+^]_i._. Incubation of Kyse-140 cells with 100 μM 2MeSATP did not alter [Na^+^]_i_, whereas application of gramicidin markedly increased [Na^+^]_i_. [Na^+^]_i_ is given as the fluorescence ratio (ΔF344/F380 nm) of the Na^+^-sensitive dye SBFI/AM. Representative single-cell tracing out of three independent experiments with 18 or more cells per experiment is shown.

For further pharmacological characterization, a possible contribution of P2X-receptors to the observed changes in [Ca^2+^]_i_ was investigated by using the P2X-receptor specific agonists 2-methylthio-ATP (2MeSATP) and α,β-methylene-ATP (α,β-meATP). P2Y_1_-, P2Y_11_-, P2Y_ADP_- and the ionotropic P2X_1–6_-receptors have been shown to be sensitive for 2MeSATP (Lambrecht, 2000). However, 100 μM 2MeSATP did not evoke any increase of [Ca^2+^]_i_ in Kyse-140 cells (Figure 4E). Even at the high concentration of 500 μM, 2MeSATP showed no [Ca^2+^]_i_-inducing effects (data not shown). However, it has been shown by others (Weidema *et al*, 2001), that activation of P2X_4_-receptors does not necessarily influence [Ca^2+^]_i_ but is implicated in alterations of intracellular sodium concentrations ([Na^+^]_i_). Therefore additional [Na^+^]_i_-measurements with 2MeSATP were performed. Nevertheless, applying 100 μM 2MeSATP on Kyse-140 cells, preloaded with the [Na^+^]_i_-sensitive dye SBFI (10 μM), did not evoke alterations in [Na^+^]_i_ ( Figure 5

). Comparable negative results were obtained by applying ATP or UTP (100 μM) (data not shown). As positive control the cells were treated with 10 μM gramicidin at the end of each experiment. Gramicidin is known to perforate the cell membrane to become sodium-permeable (Diarra *et al*, 2001). Gramicidin treatment always led to an increase of [Na^+^]_i_ (Figure 5). In line with the negative results of P2X_4_-immunocytochemistry our data suggest, that despite the mRNA expression of P2X_4_-receptors, neither protein nor functional P2X_4_ receptor expression occurred in Kyse-140 cells.

As expected by the negative results of RT–PCR for the mRNA-expression of P2X_1_ and P2X_7_ receptors, no increase in [Ca^2+^]_i_ was observed when the cells were challenged by the P2X-receptor agonist α,β-meATP, which additionally excluded a contribution of these receptor subtypes in the observed apoptotic and cell cycle arresting effects on oesophageal cancer cells. To conclude, the rank order of potency of the applied nucleotides to increase [Ca^2+^]_i_ was ATP=UTP>ATPγS>ADP=UDP>>2MeSATP=α,β-meATP. This graded efficiency of different nucleotides to increase [Ca^2+^]_i_ reflects the pharmacological pattern of P2Y_2_-receptors, which thus seems to be the only functionally expressed P2-receptor subtype in squamous oesophageal cancer cells.

**Figure 6:**
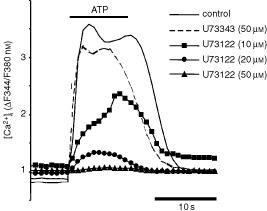
Phospholipase C pathway. P2Y_2_-receptor mediated [Ca^2+^]_i_-elevations in Kyse-140 cells are mediated by phospholipase C activation. Pre-treatment (10 min) of Kyse-140 cells with the phospholipase C inhibitor U73122 (10–50 μM) dose-dependently inhibited [Ca^2+^]_i_ elevation in response to ATP (100 μM), whereas the inactive U73122-analogue U73343 (50 μM) did not affect P2Y_2_-receptor mediated [Ca^2+^]_i_ signalling. [Ca^2+^]_i_ is given as the fluorescence ratio (ΔF340/F380 nm) of the Ca^2+^-sensitive dye fura-2. Representative single-cell tracings out of five independent experiments with 20 or more cells per incubation are shown.

P2Y_2_-receptors couple to phospholipase C via G_q/11_ proteins (Burnstock, 1997). To determine the role of phospholipase C in the signalling pathway of P2Y_2_-receptors in Kyse-140 cells, the specific phospholipase C inhibitor U73122 was used. U73122 dose-dependently inhibited the increase of [Ca^2+^]_i_ upon ATP stimulation at micromolar concentrations, whereas its inactive analogue U73343 showed little if any effect ( Figure 6

). Fifty μM U73122 completely suppressed the ATP induced [Ca^2+^]_i_-rises, confirming that in Kyse-140 cells P2Y_2_-receptors couple to phospholipase C and thereby effect [Ca^2+^]_i_-rises.

## DISCUSSION

ATP is released from malignant cells in response to chemo- or radiotherapy. Extracellular ATP is known to inhibit growth of Ehrlich ascites tumour cells, colon cancer, ovarian cancer, endometrial cancer, breast cancer, and fibroblast cell lines (Estrela *et al*, 1995; Correale *et al*, 1997; Höpfner *et al*, 1998; Katzur *et al*, 1999; Li *et al*, 2000; Schultze-Mosgau *et al*, 2000). Here we provide evidence that extracellular nucleotides induce apoptosis and cause cell cycle arrest in human squamous cancer cells of the oesophagus.

ATP and ATPγS induced a delay in the S-phase of the cell cycle. The cell cycle arresting effects of extracellular nucleotides were similar to the ones elicited by the cytostatic drugs 5-fluorouracil and cisplatin in oesophageal cancer cells (Yamane *et al*, 1999; Mastbergen *et al*, 2000). Moreover, for breast and ovarian cancer a positive correlation between S-phase fraction and the response to anticancer agents has recently been documented (Chevillard *et al*, 1996; Kolfschoten *et al*, 2000). Hence, recruitment of cancer cells to the S-phase of the cell cycle by ATP might sensitise oesophageal cancer cells to a reinforced response to established chemotherapeutic drugs. Furthermore, cyclin-dependent kinase inhibitors such as flavopiridol, a promising new class of anticancer drugs, showed enhanced cytotoxity at cells arrested in the S-phase (Shapiro and Harper, 1999). Hence, in addition to the antiproliferative action of ATP on its own, possible synergistic effects of ATP and anticancer drugs will be interesting to investigate. Accordingly, a synergistic antiproliferative effect of 5-FU and extracellular ATP has recently been described in colorectal carcinoma cells (Höpfner *et al*, 2001).

Besides its cell cycle interfering effects, ATP was shown to induce apoptosis in oesphageal cancer cells as assessed by TUNEL assays and measuring increases of caspase-3 activity. Both primary cell cultures and Kyse-140 cells were shown to undergo apoptosis upon ATP treatment. The ability of ATP to induce apoptosis via P2-receptors in a [Ca^2+^]_i_-dependent manner has been formerly demonstrated by others (Chow *et al*, 1997). On the other hand, using the hydrolysis resistant ATP derivative ATPγS, Katzur *et al* (1999) observed P2Y_2_-mediated antiproliferative but not apoptosis inducing effects in endometrial carcinoma cells. In our study both nucleotides, ATP and the ATPγS, evoked rises of [Ca^2+^]_i_, but only ATP induced apoptosis. This suggests that in addition to the P2Y_2_-receptor triggered [Ca^2+^]_i_-increase other signalling pathways may mediate apoptosis of oesophageal cancer cells in response to ATP. Interestingly, Zoeteweij *et al* (1993) showed that in hepatocytes [Ca^2+^]_i_-induced apoptosis after prolonged exposure to ATP was dependent on the generation of extracellular inorganic phosphate (P_i_), which resulted from an ecto-ATPase mediated hydolysis of the applied extracellular ATP. Hence, inorganic phosphate might be an additional signal necessary to induce apoptosis in oesophageal cancer cells. Since ATP is hydrolyzed by ubiquitously expressed ecto-ATPases and ectonucleotidases, this could explain, why the hydrolysis resistant ATP derivative ATPγS, which is additionally known to be an effective ecto-ATPase-inhibitor (Chen and Lin, 1997), failed to induce apoptosis specific caspase-3 activation in oesophageal cancer cells. This interpretation was supported by our finding that the ecto-ATPase-inhibitor ARL 67156, which diminished the generation of P_i_ by inhibiting ATP-degradation, resulted in a reduced activation of ATP induced caspase-3 activity.

Antiproliferative as well as apoptosis inducing actions of extracellular nucleotides are thought to result from (prolonged) stimulation of functionally expressed P2-receptors. While antiproliferative effects of metabotropic P2Y-receptors have been documented in various tumour models (Fang *et al*, 1992; Vandewalle *et al*, 1994; Höpfner *et al*, 1998; Katzur *et al*, 1999; Schultze-Mosgau *et al*, 2000), apoptosis inducing effects of P2Y-receptors have only been shown in MCF-7-breast cancer (Vandewalle *et al*, 1994) and colorectal carcinoma cells (Höpfner *et al*, 2001) so far. Here we provide evidence that the cell cycle arresting and apoptosis inducing actions of ATP involved the activation of P2Y_2_-receptors in oesophageal cancer cells. Expression of P2Y_2_-receptors in primary culture cells of oesophageal cancers as well as in Kyse-140 cells was revealed by both RT–PCR and by immunocytochemistry. Performing [Ca^2+^]_i_-imaging Kyse-140 cells were shown to be equally sensitive to both purine and pyrimidine nucleotides demonstrating the functionality of the expressed P2Y_2_-receptor protein. The functional expression of the P2Y_2_-receptor subtype was strengthened by the complete cross-desensitization between the two major agonists, ATP and UTP. Furthermore, the increase of [Ca^2+^]_i_ was shown to be PLC-dependent, indicating that the induction of [Ca^2+^]_i_ was mediated by G_q/11_-coupled and PLC-linked metabotropic P2Y_2_-receptors.

However, the main P2-receptor subtypes implicated in apoptosis are ionotropic P2X-receptors of the subtypes P2X_1_ and P2X_7_ (Chvatchko *et al*, 1996; Di Virgilio *et al*, 1998; Ferrari *et al*, 1999). P2X-receptors are mainly found in excitable tissues such as smooth muscles and nerves, although P2X_4_-receptors have also been reported in endocrine tissues (Ralevic and Burnstock, 1998). However, neither in primary cultures of oesophageal cancers nor in the oesophageal cancer cell line Kyse-140 mRNA expression of either P2X_1_- or P2X_7_-receptors could be detected. Only transcripts of the ionotropic P2-receptor subtypes P2X_4_ and P2X_5_ were found. So far neither P2X_4_ nor P2X_5_ has been implicated in the induction of apoptosis. Moreover, the functional expression of P2X_5_-receptors is most unlikely, since the human P2X_5_-receptors described are unable to form functional channels (North and Surprenant, 2000). Nevertheless, to examine the possible expression and functionality of P2X_4_-receptors in oesophageal cancer cells immunocytochemistry, [Ca^2+^]_i_- and [Na^+^]_i_-measurements were performed. Immunofluorescence microscopy with the specific P2X_4_-receptor antibody Ab-1 failed to yield a P2X_4_-receptor staining in either primary cultured cells or in the squamous oesophageal cancer cell line Kyse-140. Also the application of the P2X_4_-receptor-specific agonist 2MeSATP failed to alter [Ca^2+^]_i_. Since it has been reported that in osteoclasts P2X_4_-receptors do not couple to [Ca^2+^]_i_-signalling but influence [Na^+^]_i_ (Weidema *et al*, 2001), we performed [Na^+^]_i_-measurements. However, 2MeSATP induced alterations of [Na^+^]_i_ could not be detected. Thus, on the basis of our data we exclude protein and functional expression of P2X_4_-receptors in oesophageal cancer cells – despite positive RT–PCR-findings. Altogether, P2X-receptors do not appear to contribute to ATP-induced [Ca^2+^]_i_-signalling or to growth inhibition and apoptosis in Kyse-140 oesophageal cancer cells.

To ensure that the observed antiproliferative effects of extracellular nucleotides on oesophageal cancer cells were actually P2Y_2_-receptor mediated events and were not caused by unspecific effects of the applied nucleotides we also performed proliferation assays with the P2X_4_-receptor agonist 2MeSATP. Since we have excluded a functional expression of P2X_4_-receptors in the investigated oesophageal cancer cells and it is known that 2MeSATP does not activate P2Y_2_-receptors to a relevant extent (Ralevic and Burnstock, 1998), only unspecific signalling of the applied nucleotide could yield antiproliferative effects. However, no contribution of 2MeSATP on the growth of oesophageal cancer cells could be observed – even at a concentration of 500 μM. These data suggest that the induction of growth inhibition and apoptosis in oesophageal cancer cells by extracellular ATP and ATPγS was caused by the activation of P2Y_2_-receptors, but was not due to unspecific effects of these extracellular nucleotides.

To conclude, our data show that extracellular nucleotides cause cell cycle arrest and induce apoptosis in human oesophageal carcinoma cells. These actions are mediated by P2Y_2_-receptors. Interestingly, ATP has already been studied as an anti-cachexia and antiproliferative agent in advanced lung-cancer (Haskell *et al*, 1996; Agteresch *et al*, 1999). Taken together, we therefore think that P2Y_2_-receptors may qualify as promising targets for innovative treatment strategies of oesophageal cancer.
